# Prevalence and Dynamics of SARS-CoV-2 Antibodies in the Population of St. Petersburg, Russia

**DOI:** 10.1007/s44197-022-00041-9

**Published:** 2022-05-30

**Authors:** Ekaterina V. Parshina, Alexey B. Zulkarnaev, Alexey D. Tolkach, Andrey V. Ivanov, Pavel N. Kislyy

**Affiliations:** 1 Nephrology and Dialysis Department, Saint Petersburg State University Hospital, 154, Fontanka Emb., Saint-Petersburg, 198103 Russian Federation; 2 Surgical Department of Transplantology and Dialysis, M.F. Vladimirsky Moscow Regional Research Clinical Institute, 61/2, Shchepkina Str., Moscow, 129110 Russian Federation; 3 Human Genetics Department, Saint Petersburg State University Hospital, 154, Fontanka Emb., Saint-Petersburg, 198103 Russian Federation; 4 Polyclinic Department №4, Saint Petersburg State University Hospital, 154, Fontanka Emb., Saint-Petersburg, 198103 Russian Federation

**Keywords:** COVID-19, SARS-CoV-2, Antibody, Seropositivity, Seroprevalence, Quarantine

## Abstract

**Background:**

The aim of the study was to assess the prevalence of seropositive status for severe acute respiratory syndrome coronavirus 2 (SARS-CoV-2)-IgA, -IgM, and -IgG; its dynamics in connection with restrictive measures during the coronavirus disease (COVID-19) pandemic; and the quantitative dynamics of antibody levels in the population of St. Petersburg, Russia.

**Methods:**

From May to November 2020, a retrospective analysis of Saint Petersburg State University Hospital laboratory database was performed. The database included 158,283 test results of 87,067 patients for SARS-CoV-2 detection by polymerase chain reaction (PCR) and antibody detection of SARS-CoV-2-IgA, -IgM, and -IgG. The dynamics of antibody level was assessed using R v.3.6.3.

**Results:**

The introduction of a universal lockdown was effective in containing the spread of COVID-19. The proportion of seropositive patients gradually decreased; approximately 50% of these patients remained seropositive for IgM after 3–4 weeks; for IgG, by follow-up week 22; and for IgA, by week 12. The maximum decrease in IgG and IgA was observed 3–4 months and 2 months after the detection of the seropositive status, respectively.

**Conclusions:**

The epidemiological study of post-infection immunity to COVID-19 demonstrates significant differences in the dynamics of IgA, IgM, and IgG seropositivity and in PCR test results over time, which is linked to the introduction of restrictive measures. Both the proportion of seropositive patients and the level of all antibodies decreased in terms of the dynamics, and only approximately half of these patients remained IgG-positive 6 months post-infection.

**Supplementary Information:**

The online version contains supplementary material available at 10.1007/s44197-022-00041-9.

## Introduction

Since December 2019, the global community has been affected by the coronavirus disease (COVID-19) pandemic caused by severe acute respiratory syndrome coronavirus 2 (SARS-CoV-2). Due to the novelty and rapid spread of the infection, one of the challenges in control tactics has been the lack of sufficient data on the prevalence and duration of the humoral immune response. Since the first confirmed case of COVID-19, which was reported on March 2, 2020, more than 185 million polymerase chain reaction (PCR) tests have been performed in the Russian Federation to diagnose the disease [1], but studies to assess seroprevalence have not been performed. The aim of this study was to assess the prevalence of SARS-CoV-2-IgA, IgM, and IgG; its dynamics in the St. Petersburg population during the COVID-19 pandemic; and the impact of restrictive measures.

## Materials and Methods

### Study Participants

We conducted a retrospective observational study. The dataset was based on systematized information obtained from the Saint Petersburg State University Hospital laboratory database, inclusively summarizing the demographic factors, etiological categories of patients, results of SARS-CoV-2 detection by real-time PCR, and serum IgA, IgM, and IgG antibodies for SARS-CoV-2, performed from May to November 2020. Twenty-five laboratory units were evenly distributed in all districts of St. Petersburg. Since all residents of St. Petersburg were equally likely to be tested in this laboratory, we believe that the sample was representative. A total of 158,283 test results obtained from 87,067 patients were available. The number of tests performed and their characteristics are given in Table 1.

**Table 1:** Type and number of tests and the number of patients

Test	Type of test	Test	*N* tests	*N* subjects
IgA	Semi-quantitative	Euroimmun, Germany	9910	5271
IgM	Semi-quantitative	Abbott, Ireland	962	938
IgM	Qualitative	Vector-Best, Russian Federation	11,578	9732
IgG	Semi-quantitative	Euroimmun, Germany	18,869	14,165
IgG	Qualitative	Abbott, Ireland	2762	2695
PCR	Qualitative	Vector-Best, Russian Federation	114,202	73,021

*PCR* polymerase chain reaction

As shown in Table 1, antibodies were determined using a semi-quantitative or qualitative method. We established a patient's seropositive status if the qualitative test was positive or the semi-quantitative test value was greater than the threshold (≥ 1.1 for IgA and IgG, ≥ 1.4 for IgM). The semi-quantitative determination of antibodies to SARS-CoV-2 classes IgA and IgG was performed by an enzyme immunoassay using the Euroimmun SARS-CoV-2 S1 IgA/G test system (Euroimmun, Lübeck, Germany), which is based on the comparison of the optical density of control serum or the clinical sample and the optical density of the calibrator (expressed in units: optical density [OD]). The result is a numerical value (ratio) reflecting the luminescence intensity, which is thus a surrogate for the amount of antibodies of a respective class.

Some of the patients underwent the tests (at least one of the four) more than once (1–35 times). Of those with available test results, the period from the first to the last test ranged from 1 to 225 days. We analyzed the dynamics of patients’ status (seropositive/seronegative) by calendar month and by periods (weeks or months) starting from the day of the first positive test for IgA, IgM, or IgG. However, if the patient was tested repeatedly during the period, we considered the worst value as the final result: "positive" in the qualitative test or the highest value in the semi-quantitative test.

### Ethics Approval

The Biomedical Ethics Board of Saint Petersburg State University Hospital approved the study and waived the requirement for informed consent (protocol No. 03/21 from 18.03.2021). All data were de-identified pre-analysis.

### Statistical Analysis

Normally distributed quantitative data were presented as mean ± standard deviation, whereas parameters with skewed distribution were expressed as median and interquartile range (*Q*_1_–*Q*_3_). Absolute values and percentages were used to describe categorical data.

Since the semi-quantitative tests provided a numerical value, we analyzed these data as quantitative. As the observations were clustered and the matrix had missing values, we assessed the dynamics of quantitative IgA and IgG tests in patients at different weeks using a Linear Mixed-Effects Model (analysis of variance), where the fixed effect was “week” and the random effect was “patient”: lmer(IgA ~ month + (1|patient)). The analysis was performed using R v.3.6.3 (RStusio v. 1.2.5033; RStudio, Boston, MA, USA) and the “lme4” package [2]. We calculated the statistical significance of the fixed effect using Satterthwaite approximation (lmerTest package [3]) since the calculation of *p* values is not implemented in lme4 software package. *p* values < 0.05 were considered statistically significant. Pairwise comparisons were made using Tukey’s post-hoc test.

Since the assumption of homoscedasticity was not met, the Box–Cox transformation was performed (the “boxcox” function from the package “MASS” [4]). The transformed values were used in the analysis.

## Results

The patients in the study comprised 41,339 (47.5%) males and 45,728 (52.5%) females. The mean age was 40.9 ± 15.6 years and median, 40 years (Q1–Q3: 31; 52), ranging from < 1 to 99 years.

**Fig. 1:**
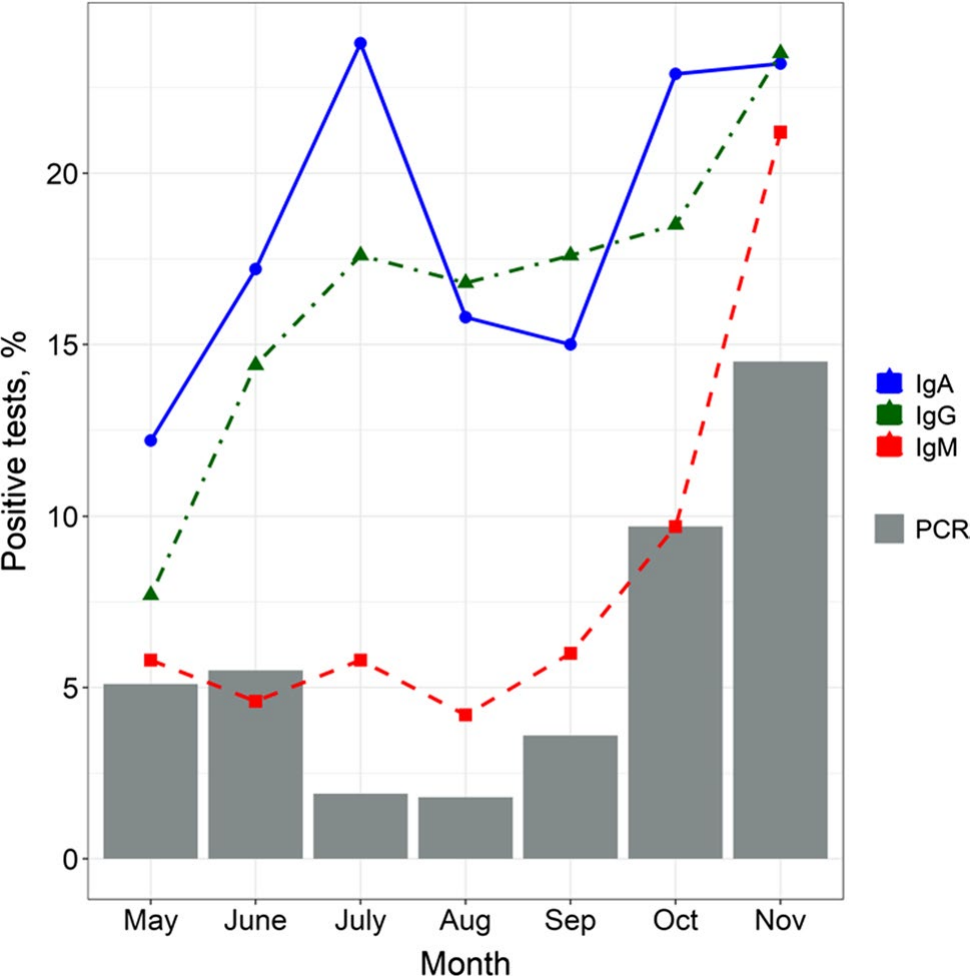
Prevalence of positive SARS-CoV-2 IgA, IgM, IgG, and PCR patients for whom each test result was available separately. If more than one result was available for a patient in a calendar month, the "worst" status (most positive) was considered. Exact estimates of positive, borderline, and negative test results are given in Appendix 1 (Tables S2-S5). PCR, polymerase chain reaction

Over the course of the study, 6.1% of individuals tested were positive for SARS-CoV-2 PCR, 17.9% for IgA, 7.2% for IgM, and 16.7% had seropositivity for IgG. Being estimated by calendar months, these positivity rates showed different trends (Fig. 1). Seroprevalence of all SARS-CoV-2 antibodies was the lowest in May 2020. The proportion of positive tests for IgA and IgG gradually increased thereafter and reached its first peak in July 2020 (23.8% and 17.6%, respectively). Seroprevalence of IgA dropped to 15% in Aug and Sept, whereas IgG seropositivity remained on the plateau during this period. Further increase in both IgA and IgG seroprevalence was observed in Oct with the second peak by Nov 2020 (23.2% and 23.5%, respectively). In contrast, IgM seropositivity remained relatively low until Sept–Oct 2020, with a sharp subsequent increase.

**Fig. 2:**
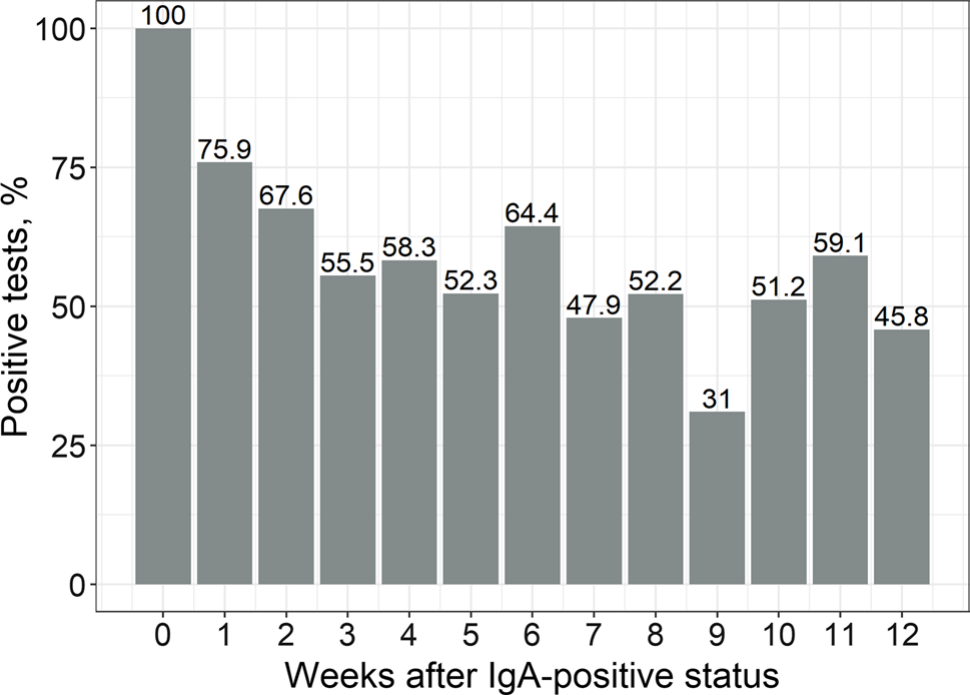
Dynamics of SARS-CoV-2 IgA seropositivity since the first positive test result. If more than one result was available for a patient during each week, the “worst” status was considered (in order: positive, doubtful, negative)

**Fig. 3:**
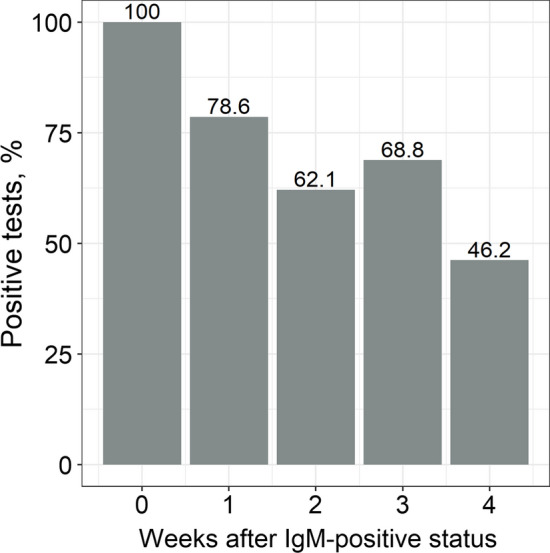
Dynamics of SARS-CoV-2 IgM seropositivity since the first positive test result. If more than one result was available for a patient during each week, the “worst” status was considered (in order: positive, doubtful, negative)

The dynamics of the serostatus since the first positive result was evaluated for each antibody type separately. Overall, all antibodies demonstrated a downward tendency over time. In particular, patients lost their seropositive status for IgM and IgA faster: only half of the patients were seropositive one month after the first positive test result (Figs. 2 and 3). However, IgA positivity persisted up to 12 weeks in approximately half of the cases, which is slightly different from the “classic” pattern of humoral immune response to viral infections.

**Fig. 4:**
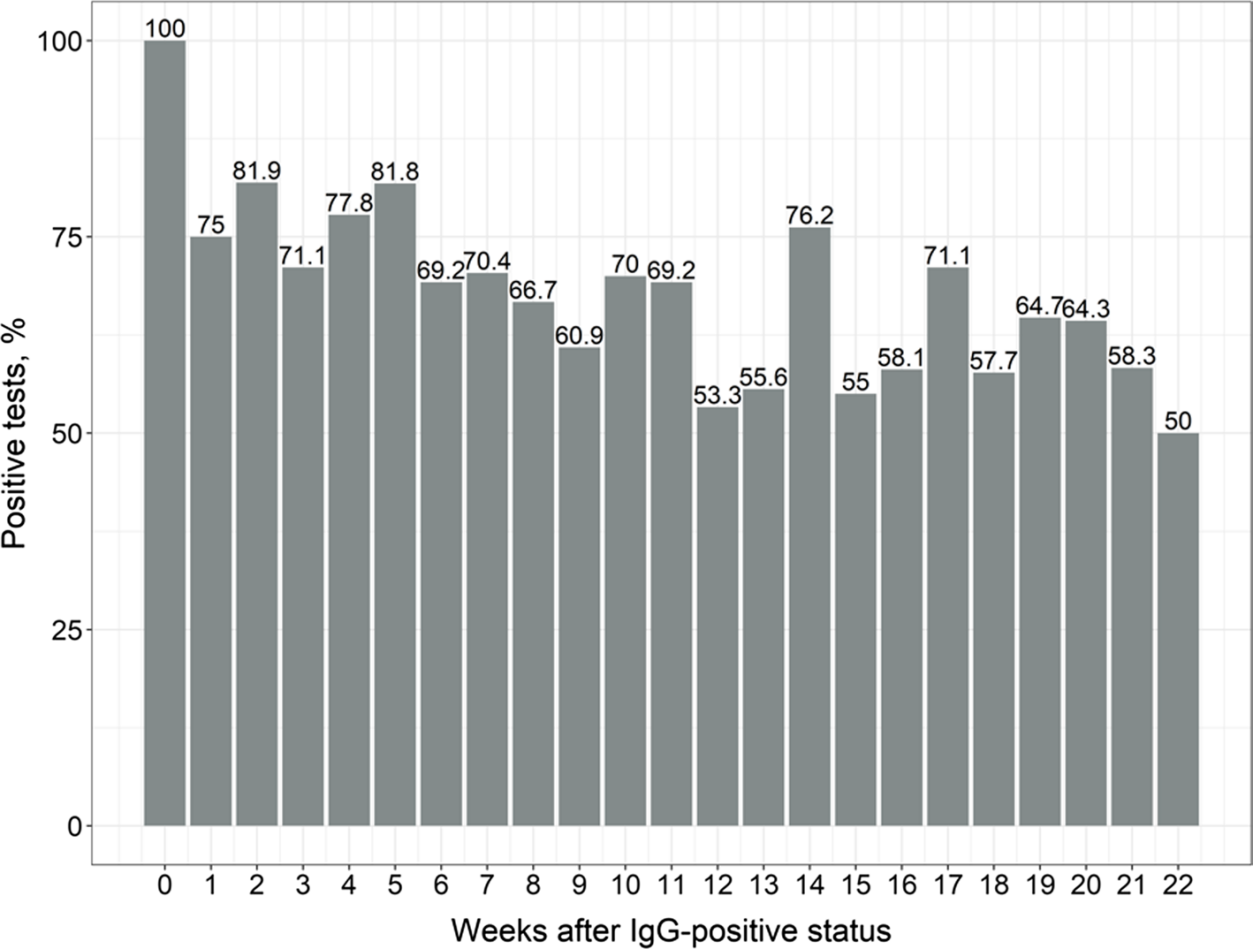
Dynamics of SARS-CoV-2 IgG seropositivity since the first positive test result. If more than one result was available for a patient during each week, the “worst” status was considered (in order: positive, doubtful, negative)

Herewith, decline in IgG seropositivity over time was less pronounced: as it can be seen from Fig. 4, more than 60% of the patients maintained seropositive status up to 12 weeks after the first positive test result, and approximately a half remained seropositive by 22 weeks post-disease.

**Fig. 5:**
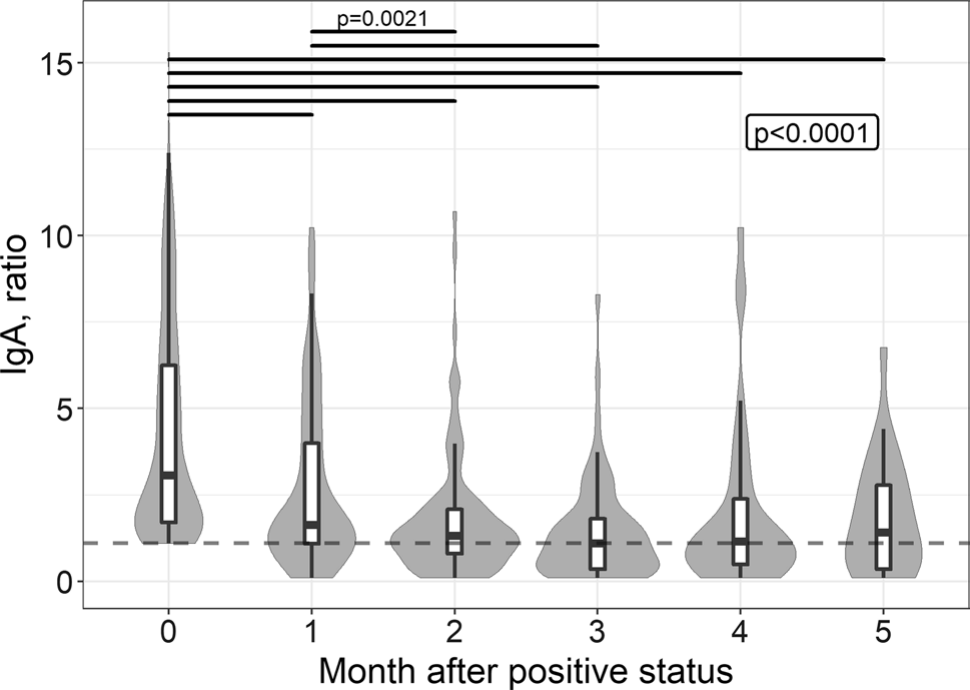
SARS-CoV-2 IgA level plotted over time, starting from the first positive test result. Native (non-transformed) data, medians, first and third quartiles are given, the shape of the background figures reflects the distribution. If more than one result was available for the patient during the month, the “worst” status (the highest test value) was considered. The dotted red line indicates the assay cut-off value (1.1). The box indicates the statistical significance of the fixed effect (“month”) in the omnibus test, with solid lines indicating statistically significant pairwise comparisons (if *p* value estimate not given, it is less than 0.0001)

**Fig. 6:**
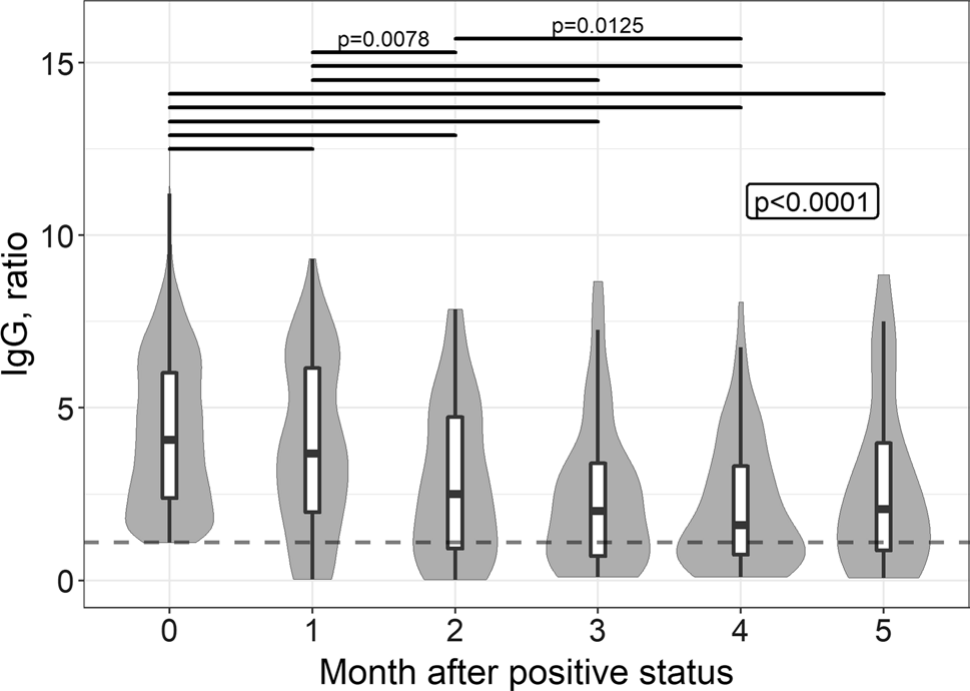
SARS-CoV-2 IgG level plotted over time, starting from the first positive test result. Native (non-transformed) data, medians, first and third quartiles are given, the shape of the background figures reflects the distribution. If more than one result was available for the patient during the month, the "worst" status (the highest test value) was considered. The dotted red line indicates the assay cut-off value (1.1). The box indicates the statistical significance of the fixed effect (“month”) in the omnibus test, with solid lines indicating statistically significant pairwise comparisons (if *p* value estimate not given, it is less than 0.0001)

For patients whose quantitative test results were available, we assessed the dynamics of IgA (Fig. 5, 1464 repeated observations were available) and IgG (Fig. 6, 2851 repeated observations were available) using mixed effect analysis of variance. Because we noted a violation of the conditions of applicability of this method, the data underwent a pre-analysis Box-Cox transformation. Figures 5 and 6 show the native data. Both IgA and IgG levels demonstrated a statistically significant decrease from the baseline to the followed months of observation. However, for IgA this difference became not significant after 2 months of follow-up, i.e. the greatest drop occurred during this period. Specific IgG did not decrease to undetectable level in most of the patients; nevertheless, a statistically significant drop was observed during 4 months from the first positive test result.

As few repeated semi-quantitative IgM test results were available, we present only the estimate at the time of the first seropositive status: 4.875 [2.528; 10.83], ranging from 1.49 to 51.71 conventional units.

## Discussion

To the best of our knowledge, this study is the most extensive assessment of the seroprevalence and dynamics of the seropositive status for different types of SARS-CoV-2 antibodies during the COVID-19 pandemic in the Russian Federation. The general lockdown was officially introduced in the country on March 30, 2020. From May 12, 2020, there was a gradual lifting of restrictions; in July to August, most restrictions were lifted in all regions of the Russian Federation. Since the beginning of the pandemic, St. Petersburg has been leading in the number of reported cases [1]; thus, the prevalence and dynamics of antibody response are representative and can reflect the accumulated share of COVID-19 cases in the general population. Figure 1 shows that the proportion of positive PCR tests detected was at an all-time low in July and August, while the proportion of positive tests for anti-SARS-CoV-2 IgM was unchanged from May to August 2020. This indicates that the incidence of COVID-19 during this period was low, which appears to be the result of a timely lockdown introduction. In Italy, the first country in Western Europe faced with COVID-19, the virus has diffused rapidly before the lockdown was imposed. As a result, according to the cross-sectional study on the SARS-CoV-2 seroprevalence in the Bergamo province, the cumulative prevalence turned out to be overwhelmingly high: specific antibodies were found in 38.5% of the study population [5]. On the contrast, we found 14.5% prevalence for positive IgG to the end of the first wave of the disease (July 2020), which is similar to that reported for the other large cities, including Madrid with around 10% [6], Geneva with 10.9% [7], and London with 12% [8]. At the same time, high seroprevalence (23.6%) was observed in New York after the first wave despite the quarantine introduced [9], and can be explained by possible low adherence to public health interventions due to socioeconomic factors and wide-spread misinformation about the COVID-19 pandemic [10, 11].

Based on the data obtained in the present study, we can confidently judge the effectiveness of the restrictive measures taken in our country at the very beginning of the pandemic. A steady increase of the anti-SARS-CoV-2-IgG prevalence from May to July 2020 was most likely due to a seroconversion during the first “wave” of the disease. The disease situation remained stable until September to October 2020, when the proportion of both PCR-positive and IgM/IgG seropositive results started to rise sharply. It is possible that the tactic of reintroducing restrictive measures could curb the severity of the second wave of disease, but a decision to reintroduce the lockdown has not been made.

Early epidemiological studies from China demonstrated the positive impact of lockdown on COVID-19 spread. Ji et al. found that lockdown resulted in flattening the epidemic curve in Huangsi, China [12]. In line with these results, Lau et al. showed decreased growth rate and increased doubling time of new COVID-19 cases after lockdown implementation in Wuhan [13]. Being a highly-effective measure, quarantine should be considered as a prudent advice for a 'pandemic preparedness plan' for the future.

Quarantine is crucial but not the only strategy to contain the spread of COVID-19. A related set of papers provides effectiveness of social distancing, testing strategies, contact tracing, travel-related control measures and limits on public gatherings in limiting spread [14–17]. Many of these measures have also been implemented in our country, which could influence the results of the present study. Data from mathematical modelling studies suggests that other preventive strategies should be implemented in combination with quarantine to raise policy effectiveness [18, 19].

Approximately half of patients who were anti-SARS-CoV-2-IgA-positive maintained this status after 3 months of follow-up. Similar results were demonstrated in a study by Seow [20]. However, in a study by Iyer et al. the median reverse seroconversion of IgA was 71 days from the onset of symptoms [21]. IgA is a secretory Ig produced by viral contact with mucous membranes and is responsible for local immunity. Thus, it can be stated that local immunity persists for up to 3 months in half of those exposed to the virus. Not all of those exposed to the virus manifested the disease: Fig. 1 shows that the dynamics of IgA and IgM prevalence are practically unrelated. At the same time, the increase in the proportion of positive anti-SARS-CoV-2-IgA tests was observed at the very beginning of the second “wave”, slightly outpacing the increase in the number of positive IgM and IgG tests. It is possible that the increase in the proportion of IgA seropositive tests can “predict” a subsequent increase in the number of patients (the next “wave”), which is a promising topic for further study.

It should be noted that the amount of IgA is still decreased in the dynamics and remains high only in some patients (Fig. 5). Similar long-term inter-patient variability in the IgA level after natural COVID-19 disease has been reported in other studies [20, 22, 23]. The exact etiological category in individuals demonstrating prolonged IgA persistence that’s yet to be elucidated. These may be people whose occupations involve frequent social contact (health care workers, educators, and service providers). There is evidence that IgA is the only independent predictor of the severity of COVID-19 [24], and the duration of its persistence may be determined by the initial severity of the disease. In contrast to IgA, IgM exhibits a classic downward trend and is detectable in only half of the baseline positive patients after 4 weeks of follow-up. Differences in the kinetics of IgA and IgM have previously been noted by other researchers, although the observation period was short (40 days) [25].

The dynamics of anti-SARS-CoV-2-IgG as an antibody responsible for long-term protection against re-infection is of great interest. As shown in Fig. 4, half of the patients lose their seropositive status by 6 months. The amount of IgG also decreases significantly with each month, as has also been described by other researchers [26, 27]. However, some data suggest that the IgG persists for longer periods of time. For example, in a large observational study that included laboratory data from 39,000 subjects, the rate of IgG seropositivity to the SARS-CoV-2 spike protein was 87.8% by 300 days of follow-up [28]. However, due to the long follow-up duration, this data should be interpreted with caution, as re-infection cannot be excluded. Prognostic models based on different patterns of antibody dynamics predict the duration of IgG persistence, with a wide range from 40 d to a few decades [29]. Although there was a statistically significant decrease in the IgG from the baseline to the subsequent months of follow-up, Fig. 6 shows that the maximum decrease occurs 3–4 months after the infection, which appears to be the optimal time for vaccination.

### Study Limitations

First, the study was retrospective in its nature. Second, we had access to data from only one laboratory. Nevertheless, the sample was randomly obtained from the target general population, which allows sufficient objectivity. Third, in this study, we did not take into account the fact that some patients may have been infected repeatedly. The reason for it is our assumption that the proportion of re-infections was not high in the analyzed time interval. Fourth, we only had information on laboratory test results, not on the clinical course of the disease and the need for hospitalization. Therefore, we had to restrict our study to the terms “positive status” but not “ill”. Fifth, probably the main limitation of the study is the use of different antibody assays. To overcome this limit, we defined patients’ seropositive status using manufacturer’s recommended cut-off values for strictly positive test results. “Doubtful” results for both qualitative and quantitative tests were interpreted as negative. In addition, we used only the Euroimmun immunoassay results in the assessment of qualitative antibody dynamics.

As it can be seen from the Table 1, the results of repeated analyzes were not available for all patients. It is likely that the retests were performed on people who had a specific reason for it. Thus, it may lead to a false overestimation of the proportion of seropositive individuals. However, in the context of the present study, it means that seropositivity is being lost even faster than it seems.

In conclusion, this epidemiological study of post-infection immune response to COVID-19 demonstrates significant differences in the dynamics of seropositivity for IgA, IgM, and IgG and PCR test results over time, with a clear link to the introduction of restrictive measures. Both the proportion of seropositive patients and the level of antibodies of all classes decreased over time, with only half of patients remaining IgG-positive by 6 months post-infection.

## Supplementary Information

Below is the link to the electronic supplementary material.

Supplementary file1 (DOCX 27 KB)

## Data Availability

The data of the study, including the code used in the analyses, are available from the corresponding author upon reasonable request.

## Abbreviations

SARS-CoV-2: Severe acute respiratory syndrome coronavirus 2
COVID-19: Coronavirus disease
PCR: Polymerase chain reaction
