# Supplementary material for: Prevalence and Dynamics of SARS-CoV-2 Antibodies in the Population of St. Petersburg, Russia
Source: J Epidemiol Glob Health. 2022 May 30;12(2):206–13. doi: 10.1007/s44197-022-00041-9 (PMC9148942; doi:10.1007/s44197-022-00041-9)
Supplement: Supplementary file 1 — Supplementary file1 (DOCX 27 KB) [file 44197_2022_41_MOESM1_ESM.docx]

**APPENDIX 1.**

Тable S2. IgA serological status of the patients. If more than one result was available for a patient during each month, the "worst" status was considered (in order: positive, doubtful, negative).

| IgA | May | June | July | August | September | October | November | All |
| --- | --- | --- | --- | --- | --- | --- | --- | --- |
| Positive | 155 (12,2%) | 356 (17,2%) | 297 (23,8%) | 143 (15,8%) | 127 (15%) | 125 (22,9%) | 119 (23,2%) | 1322 (17,9%) |
| Doubtful | 33 (2,6%) | 72 (3,5%) | 57 (4,6%) | 38 (4,2%) | 37 (4,4%) | 29 (5,3%) | 24 (4,7%) | 290 (3,9%) |
| Negative | 1081 (85,2%) | 1643 (79,3%) | 895 (71,7%) | 724 (80%) | 683 (80,6%) | 392 (71,8%) | 370 (72,1%) | 5788 (78,2%) |
| All | 1269 (100%) | 2071 (100%) | 1249 (100%) | 905 (100%) | 847 (100%) | 546 (100%) | 513 (100%) | 7400 (100%) |

Table S3. IgM serological status of the patients. If more than one result was available for a patient during each month, the "worst" status was considered (in order: positive, doubtful, negative).

| IgM | May | June | July | August | September | October | November | All |
| --- | --- | --- | --- | --- | --- | --- | --- | --- |
| Positive | 13 (5,8%) | 174 (4,6%) | 163 (5,8%) | 57 (4,2%) | 68 (6%) | 153 (9,7%) | 234 (21,4%) | 862 (7,2%) |
| Doubtful | 6 (2,7%) | 45 (1,2%) | 59 (2,1%) | 18 (1,3%) | 12 (1,1%) | 32 (2%) | 5 (0,5%) | 177 (1,5%) |
| Negative | 204 (91,5%) | 3544 (94,2%) | 2584 (92,1%) | 1286 (94,5%) | 1062 (93%) | 1394 (88,3%) | 853 (78,1%) | 10927 (91,3%) |
| All | 223 (100%) | 3763 (100%) | 2806 (100%) | 1361 (100%) | 1142 (100%) | 1579 (100%) | 1092 (100%) | 11966 (100%) |

Table S4. IgG serological status of the patients. If more than one result was available for a patient during each month, the "worst" status was considered (in order: positive, doubtful, negative).

| IgG | May | June | July | August | September | October | November | All |
| --- | --- | --- | --- | --- | --- | --- | --- | --- |
| Positive | 127 (7,7%) | 728 (14,4%) | 680 (17,6%) | 308 (16,8%) | 250 (17,6%) | 544 (18,5%) | 563 (23,5%) | 3200 (16,7%) |
| Doubtful | 15 (0,9%) | 63 (1,2%) | 38 (1%) | 29 (1,6%) | 27 (1,9%) | 63 (2,1%) | 51 (2,1%) | 286 (1,5%) |
| Negative | 1504 (91,4%) | 4257 (84,3%) | 3152 (81,4%) | 1493 (81,6%) | 1140 (80,5%) | 2330 (79,3%) | 1785 (74,4%) | 15661 (81,8%) |
| All | 1646 (100%) | 5048 (100%) | 3870 (100%) | 1830 (100%) | 1417 (100%) | 2937 (100%) | 2399 (100%) | 19147 (100%) |

Тable S5. PCR-status of the patients. If more than one result was available for a patient during each month, the "worst" status was considered (in order: positive, doubtful, negative).

| PCR | May | June | July | August | September | October | November | All |
| --- | --- | --- | --- | --- | --- | --- | --- | --- |
| Positive | 743 (5,1%) | 667 (5,5%) | 225 (1,9%) | 193 (1,8%) | 454 (3,6%) | 1529 (9,7%) | 1700 (14,5%) | 5511 (6,1%) |
| Doubtful | 95 (0,7%) | 1 (0%) | 0 (0%) | 0 (0%) | 0 (0%) | 0 (0%) | 0 (0%) | 96 (0,1%) |
| Negative | 13678 (94,2%) | 11452 (94,5%) | 11838 (98,1%) | 10795 (98,2%) | 12036 (96,4%) | 14301 (90,3%) | 10011 (85,5%) | 84111 (93,8%) |
| All | 14516 (100%) | 12120 (100%) | 12063 (100%) | 10988 (100%) | 12490 (100%) | 15830 (100%) | 11711 (100%) | 89718 (100%) |

PCR, polymerase chain reaction
